# A vignette study of mental health literacy for binge-eating disorder in a self-selected community sample

**DOI:** 10.1186/s40337-023-00795-y

**Published:** 2023-05-04

**Authors:** Kayla B. Hollett, Jenna M. Pennell, Jacqueline C. Carter

**Affiliations:** Department of Psychology, Memorial University of Newfoundland, 230 Elizabeth Avenue, St. John’s, NL A1C 5S7 Canada

## Abstract

**Background:**

Mental health literacy has implications for mental disorder recognition, help-seeking, and stigma reduction. Research on binge-eating disorder mental health literacy (BED MHL) is limited. To address this gap, our study examined BED MHL in a community sample.

**Method:**

Two hundred and thirty-five participants completed an online survey. Participants read a vignette depicting a female character with BED then completed a questionnaire to assess five components of BED MHL (problem recognition, perceived causes, beliefs about treatment, expected helpfulness of interventions, and expected prognosis).

**Results:**

About half of participants correctly identified BED as the character’s main problem (58.7%). The most frequently selected cause of the problem was psychological factors (46.8%) and a majority indicated that the character should seek professional help (91.9%). When provided a list of possible interventions, participants endorsed psychologist the most (77.9%).

**Conclusions:**

Compared to previous studies, our findings suggest that current BED MHL among members of the public is better, but further improvements are needed. Initiatives to increase knowledge and awareness about the symptoms, causes, and treatments for BED may improve symptom recognition, help-seeking, and reduce stigma.

**Supplementary Information:**

The online version contains supplementary material available at 10.1186/s40337-023-00795-y.

## Abstract (summary)

Ensuring that people have accurate knowledge about eating disorders is important to reduce stigma and improve access to treatment. We conducted a study to explore what people know about binge-eating disorder mental health literacy (BED MHL). Two hundred and thirty-five participants read a vignette about an adult woman with BED and then completed an online survey to assess their knowledge. Most participants correctly identified BED as the main problem and identified appropriate causes and treatments. Our results help clarify what people know and what they do not know about BED, which can inform programs that are designed to reduce stigma and improve access to care.

**Supplementary Information:**

The online version contains supplementary material available at 10.1186/s40337-023-00795-y.

## Introduction

Mental health literacy (MHL) refers to public knowledge and understanding about mental disorders [1]. Studies show that increased MHL is associated with improved problem recognition, better help-seeking attitudes, and reduced stigma [2–4]. As such, increasing MHL is an important public health priority. Moreover, MHL is important for stigma reduction because it represents a policy variable that can be targeted directly to alter stigma [5]. To date, considerable research has focused on the consequences of mental disorder stigma, but less research has examined MHL for mental disorders, and research on MHL for eating disorders is particularly lacking [6]. Among the most common eating disorders, binge eating disorder (BED) has received the least MHL research attention [7].

BED is a serious eating disorder characterized by recurrent episodes of binge eating in the absence of extreme weight control behaviour such as purging [8]. Episodes of binge eating occur when an individual consumes an objectively large amount of food for the context, typically in a discrete time frame, while experiencing a sense of loss of control [8]. The disorder is associated with significant distress, high rates of psychiatric comorbidity [9–11], medical problems [12], and poor quality of life [13, 14]. While BED is the most common eating disorder affecting at least 2.8% of the population [11, 15], it remains underrecognized and undertreated [16] with up to 90% of people not receiving evidence-based treatment [11, 17]. Moreover, individuals with BED face considerable stigma in that they tend to be perceived as lacking self-discipline and are often blamed for their condition [18–20].

Research to date has provided preliminary evidence that the public have poor BED MHL. For example, O’Connor and colleagues [21] found that BED was the only condition of several mental and physical conditions depicted in vignettes that all participants failed to identify using an open-ended question, indicating a low ability to recognize the disorder (*N* = 290 adolescents). In another study, Anderson and colleagues [22] found that 61.9% of a sample of adolescents (*N* = 1666) were unable to correctly identify BED in a vignette and also viewed BED as less severe than bulimia nervosa. Further, McNicholas and colleagues [23] found that 80.8% of healthcare professionals failed to recognize BED in a vignette and were less likely to identify BED than bulimia nervosa, major depressive disorder, and type 1 diabetes. Taken together, these findings suggest that adolescents and healthcare professionals are not good at identifying BED from written descriptions.

In a study conducted with the general population, Mond and Hay [24] found that 88.3% of participants failed to identify BED as the correct diagnosis for a vignette character. Moreover, participants endorsed “Getting out and about more/finding some new hobbies” as the most suitable treatment option for the character’s problem. This study had some important strengths, namely the use of a nationally representative sample of 1034 participants aged 15–94. However, a limitation of the study was that the vignette character was described as “severely obese”, which may have influenced participant responses given that obesity is highly stigmatized [25, 26]. Second, the vignette description included details not pertinent to a diagnosis of BED (e.g., thoughts of engaging in weight control through exercise or laxatives). Vignette descriptors that are not diagnostic features of BED may have distracted participants from important information such as the description of the character’s binge eating episodes. Finally, considerable time has passed since Mond and Hay [24] published their work in 2008, and BED has since received recognition in the Diagnostic and Statistical Manual of Mental Disorders, 5th edition [8]. Therefore, it is possible that public knowledge about BED has changed, and a current assessment of BED MHL among members of the public is needed.

The aim of our study was to assess current BED MHL using a vignette-survey design while addressing some limitations of previous studies. Namely, we based our vignette description of BED on DSM-5 diagnostic criteria while excluding extraneous information that could distract from BED recognition (e.g., describing the character’s body weight or unrelated dieting behaviour). Additionally, we updated previous MHL questionnaires to better assess domains of MHL relevant for BED including participants’ ability to identify the main problem, their knowledge about likely causes of the problem, their understanding of the need for treatment, their ability to identify helpful interventions, and their ability to anticipate a likely prognosis if treatment were received.

Additionally, studies suggest that sociodemographic factors such as gender, age, level of education, and personal experience with an eating disorder may influence problem recognition and opinions about help-seeking for eating disorders [20, 22, 24]. Based on preliminary findings from previous studies on eating disorders, we hypothesized that being female and being younger in age would be associated with improved ability to correctly identify BED in the vignette [22, 24, 27]. We also hypothesized that being female, having a higher level of education, and having personal experience with an eating disorder would be associated with participants’ recommendation that the vignette character should seek professional help [20, 22, 24, 28, 29].

## Method

### Participants and procedure

Two-hundred and forty-four participants were recruited through advertisements posted on social networking sites (e.g., Facebook groups in Canada) and in public locations throughout St. John’s, Canada. The advertisements indicated that participants would complete an online survey on “opinions about eating behaviours” and did not mention BED or MHL specifically. Participants accessed the survey via Qualtrics by clicking on a link or emailing a research assistant. Upon accessing the survey, participants were presented with a consent form, demographic questions, a vignette, a MHL questionnaire, and a feedback sheet. Participants were excluded if they were less than 18 years old (*n* = 3), spent less than seven seconds on the vignette screen (*n* = 4), or did not complete any MHL questions (*n* = 2). The final sample (*N* = 235) was 85.1% female with a mean age was 37.6 years (range: 18–75 years). Most participants identified as Caucasian (95.7%), followed by Indigenous (2.6%), and other (1.6%). Most completed some post-secondary education (87.5%), and 8.1% self-reported having a prior eating disorder diagnosis. Participants were invited to enter a draw for one of two $50 gift cards as a token of appreciation. This study was approved by the Memorial University Interdisciplinary Committee on Ethics in Human Research.

### Materials

A vignette was developed with reference to best practice recommendations [30, 31] and previous vignettes used in eating disorder research [18, 20, 24, 32]. The vignette (see Additional file 1) described a 30-year-old woman named Jane who had BED as per DSM-5 criteria [8]. The vignette did not mention Jane’s weight, the term “binge eating”, or reveal that she had a diagnosis of BED.

The MHL questionnaire (see Additional file 1) was modelled from previous MHL questionnaires [24, 33–36]. The first question was, “Please indicate which weight category you think Jane likely belongs to” to assess beliefs about weight associated with binge eating. MHL questions followed to assess: (1) ability to identify the main problem; (2) perceived causes of the problem; (3) perceived need for treatment; (4) perceived helpfulness of interventions; and (5) perceived prognosis. The questions were ordered from general (e.g., “Do you think Jane has a problem?”) to specific (e.g., “What do you think is the primary *cause* of Jane’s problem?”). Following this, participants were asked if they currently or have previously had a problem similar to the character. Lastly, participants were informed that the character had BED and were then asked several direct questions (e.g., “Do you think binge-eating disorder is an official mental disorder?”). For each MHL question, participants were given several options to select from including, “Other (please specify)”, “Not sure”, “None of the above”, and “Not applicable (I do not think Jane has a problem)” to limit biased responding.

### Statistical analyses

Analyses were conducted using Jamovi Version 1.1.9.0. Descriptive statistics were used to assess responses to the MHL questions. Chi Squared tests of independence and Pearson correlations were used to examine relationships between demographic variables and participants’ ability to identify BED as well as their help-seeking recommendations for the vignette character. A Fischer’s exact test was conducted (due to an expected cell frequency < 5) to examine the relationship between personal experience with an eating disorder and opinions about help-seeking. A Bonferroni-corrected alpha level of 0.01 (0.05/5) was used for these analyses.

## Results

### Perceived character weight

When asked, “Please indicate which weight category you think Jane likely belongs to”, the vast majority of participants characterized Jane as either overweight or obese (90.1%) followed by the recommended weight range (7.8%), and the underweight weight range (2.2%).

### Mental health literacy

When asked “Do you think Jane has a problem?” most participants responded “Yes” (88.9%), followed by “Not sure” (10.2%), and “No” (0.9%). When asked, “How would you describe Jane’s problem?”, about half of the sample (49.5%) responded that Jane had a mental health problem (see Table 1). Next, when asked “What would you say is Jane’s *main* problem?”, more than half of participants (58.7%) selected BED (see Table 2). To assess perceived causes participants were asked, “What do you think is the *primary cause* of Jane’s problem?” and the most common response was “Psychological factors” (46.8%), followed by “Not sure” (26.8%) (see Table 3). Regarding perceived need for treatment, participants were asked, “Do you think that Jane should seek professional help for her problem?” and the majority of participants selected “Yes” (91.9%). When asked to indicate which person-based, therapy-based, and medication-based interventions they believed would be most helpful, the most common response for each category was, “Psychologist” (77.9%), “Other psychotherapy” (50.2%), and “Not sure” (35.3%), respectively (see Table 4 for the top five responses in each category). Regarding the perceived helpfulness of receiving treatment, participants were asked, “What do you think Jane’s likely prognosis would be if she received treatment?” and the most common response was “She would have a full recovery, but problems might reoccur” (70.6%) (see Table 5).

**Table 1:** Participant responses to, “How would you describe Jane’s problem?”

Mental health problem	51.1%
Both a physical and mental health problem	42.6%
Other	3.8%
Physical health problem	1.7%
Don’t think Jane has a problem	0.9%

**Table 2:** Participant responses to, “What would you say is Jane’s *main* problem?”

Binge eating disorder	58.7%
Not sure	13.6%
An eating disorder, but not anorexia or bulimia	6.8%
Depression	6.4%
Anxiety	4.7%
Other	4.3%
Bulimia nervosa	3.0%
Obesity/overweight	1.7%
Anorexia nervosa	0.9%

**Table 3:** Participant responses to, “What do you think is the *primary cause* of Jane’s problem?”

Psychological factors (e.g., self-esteem)	46.8%
Not sure	26.8%
Social factors (e.g., upbringing, life stressors)	20.4%
Other (please specify)^a^	5.1%
Biological factors (e.g., genetics)	0.9%

^a^Participants who selected “Other” were asked to specify. There were twelve responses, nine of which suggested that a combination of psychological, social, and biological factors contributed to Jane’s problem, representing 3.8% of the total sample.

**Table 4:** Top five participant responses to, “If Jane did receive help, which of the following [person-based, therapy-based, medication-based] interventions do you believe would be most helpful?”

*Person-based*	
Psychologist	77.9%
Self-help support group	48.9%
Dietician	46.4%
Family doctor	32.8%
Family member or close friend	18.7%
*Therapy-based*	
Other psychotherapy	50.2%
Behavioural weight loss and/or exercise program	44.7%
Cognitive behavioural therapy	42.6%
Family counselling/therapy	38.7%
Using a self-help treatment manual or self-help book	25.1%
*Medication-based*	
Not sure	35.3%
Antidepressant medication	29.4%
Vitamins and minerals	28.1%
Antianxiety medication	24.3%
Herbal medicines/tonics	13.6%

**Table 5:** Participant responses to, “What do you think Jane’s likely prognosis would be if she received an appropriate treatment?”

Full recovery, but problems might reoccur	70.6%
Not sure	18.3%
Partial recovery	5.5%
Full recovery with no further complications	5.1%
Don’t think Jane has a problem	0.4%

### Participants’ experience of a similar problem

When asked, “In the past, have you experienced something similar to Jane’s problem?”, over half the sample indicated that they believed they have experienced a similar problem to Jane in the past (54.9%). When asked, “Do you believe you might currently have a problem similar to Jane’s problem?”, nearly a third of participants reported that they believe they currently have a problem similar to Jane’s (32.9%).

After being informed that Jane has BED, participants were asked, “Have you ever heard of binge-eating disorder?” and the vast majority of participants responded “Yes” (95.3%). When asked “Do you think binge-eating disorder is an official mental disorder?”, participants selected “Yes” most often (77.4%), followed by “Not sure” (20.4%), and “No” (2.1%). When asked, “How common do you think binge-eating disorder is in the general adult population?” 42.2% of participants indicated more than 15% of the population, 40% of participants indicated between 5 and 15% of the population, and 17.9% of participants indicated 5% or less of the population.

### Sociodemographic variables and mental health literacy

Females were approximately twice as likely than males to correctly identify BED in the vignette (61% correct among females vs. 45% correct among males), but this difference was not significant, *χ*² (1, *n* = 231) = 2.781, *p* = .095, *OR* = 1.899. Additionally, females were approximately twice as likely as males to recommend that the character seek professional help (93% recommendation among females vs. 87% among males), but this difference was also not significant, *χ*² (229, 1) = 1.259, *p* = .2618, *OR* = 1.947. Regarding age, there was a significant moderate negative correlation between age and participants’ ability to correctly identify BED in the vignette, *r* = − .257, *p* < .01, indicating that younger participants were better able to recognize BED in the vignette than older participants. Regarding education, there was no significant correlation between level of education and participants’ belief that the character should seek professional help, *r* = .063, *p* = .340. Regarding personal experience, participants with personal experience were approximately nine times more likely to recommend that the character seek professional help compared to participants without personal experience (100% recommendation among those with personal experience vs. 90.1% among those without), although this difference was not statistically significant, *p* = .051, *OR* = 9.330.

## Discussion

This study assessed BED MHL among a self-selected sample of adults from the general population, primarily females. Overall, we found better BED MHL in our sample compared to previous studies. In terms of problem recognition, 58.7% of participants successfully identified BED as the most likely problem, which contrasts with lower problem recognition rates of 11.7% and 19.2% observed in prior studies [23, 24]. These differences could be explained by methodological differences across studies, such as differences in vignettes, or they could be explained by differences in study samples. For example, we did not mention the character’s weight in our vignette, making it less likely for participants to confuse BED with obesity. Also, about one third of our participants reported having a problem similar to the vignette character, which is a high proportion compared to previous samples [24]. It could be the case that individuals with personal experience with binge eating were drawn to participate in our study, which was advertised as a study on opinions about eating behaviours. In turn, the sample we recruited may have been more likely to identify and have knowledge about BED. Moreover, we found that being younger and female were associated with better BED recognition. While previous findings on age and BED recognition are conflicting [24, 27, 28], studies have found that females tend to be more knowledgeable about mental disorders [22, 37]. Taken together, these findings suggest that BED MHL initiatives could target groups of people who many have less knowledge on BED (e.g., people with less education, older adults, males). However, more research is needed to better understand how members of the general public understand BED, and researchers should continue to explore relationships between BED MHL and demographic characteristics to better understand who would benefit most from MHL initiatives.

Regarding perceived causes, 46.8% of participants selected “Psychological factors” as the primary cause of the character’s problem, suggesting that many understood that BED is related to mental well-being [38, 39]. However, 26.8% of participants selected “Not sure”, suggesting that many were not able to characterize BED as a mental health problem. Interestingly, participants selected “Biological factors” as the least likely cause (0.9%), which is consistent with previous findings [40]. However, the belief that biological factors do not play a causal role in the development of BED could reflect the misconceptions that people with BED are to blame for their behaviour and lack self-discipline [18, 20]. These findings point to the importance of highlighting the biopsychosocial nature of BED in MHL initiatives.

Regarding knowledge about help-seeking and interventions, most participants (91.9%) agreed that the vignette character should seek professional help. Additionally, most (70.6%) reported that if the vignette character did receive treatment, “She would have a full recovery, but problems might reoccur”. These findings are indicative of good MHL given that, while BED symptoms improve with treatment [41], relapse can occur [42]. In terms of interventions, most participants reported that seeing a psychologist (77.9%) would be most helpful person-based intervention for the character. In line with this, participants commonly selected psychotherapies such as cognitive behavioural therapy (42.6%) and family counselling (38.7%) as suitable interventions [41]. While we do not expect most members of the public to be highly familiar with BED treatments, BED MHL initiatives can include information on what is an appropriate versus an inappropriate intervention.

In terms of demographic variables related to help-seeking knowledge, females were two times more likely than males to recommend that the character seek professional help and participants with personal experience were nine times more likely than those without personal experience. Despite these moderate to large effect sizes, these differences were not statistically significant, which could reflect a lack of statistical power given our sample of 235 participants. Research would benefit from an in-depth examination of the sociodemographic characteristics related to BED MHL in a larger sample.

Given that our sample was largely female with a majority endorsing a similar problem at some point in their life, it is both surprising and encouraging that most participants agreed that the vignette character should seek help for her problem. In a vignette study by Mond and colleagues [43] on MHL for bulimia nervosa, female participants who were high risk or symptomatic for bulimia were more likely to indicate that they would *not* seek help if they had a problem like the vignette character because they would not want anyone to know, and symptomatic participants were more likely to believe that someone with bulimia would face discrimination. In contrast, our finding on help-seeking suggests that most people view binge eating as a valid health problem warranting treatment. In fact, individuals with lived experience in our sample were nine times *more* likely to recommend help-seeking. Today, BED MHL initiatives should incorporate the voices of individuals with lived experience to encourage help-seeking and reduce stigma associated with this disorder.

### Strengths, limitations, and future directions

To our knowledge, this is the first study to examine BED MHL since 2008 [24]. A primary strength was that our vignette did not include mention of the character’s body size, which reduced the possibility that weight stigma impacted our results [44, 45]. We also used DSM-5 criteria in our description and excluded symptoms that do not characterize BED (e.g., thoughts of compensatory behaviours). Additionally, we examined previous MHL questionnaires and removed options less relevant to eating behaviour (e.g., “Assertiveness or social skills training” was removed as a treatment option).

While our study provides a timely update to the existing BED MHL literature, there are some limitations. First, our sample consisted primarily of white, female participants. Future studies should recruit samples that are more diverse in gender and ethnicity/race, or participants that are underrepresented in this literature (e.g., males). Related to this, more than half of our sample (54.9%) reported having experienced a similar problem in the past, and one third (32.9%) reported currently having a similar problem. These percentages could reflect the fact that many people experience difficulties with overeating [10] or a tendency for individuals with eating difficulties to participate in studies about eating. This imposes an important restriction on the generalizability of our findings as our sample may have more knowledge about eating disorders than a random sample. Additionally, our use of a female vignette character could be considered a limitation because a significant proportion of individuals with BED are male [10, 16]. While we opted to use a female character to allow for comparisons with existing studies, responses to MHL questions could vary with character gender. More research is needed to examine vignette character demographic characteristics in relation to BED MHL. Lastly, the use of a multiple-choice format in our MHL questionnaire could have increased the likelihood of biased responding from participants. Future studies would benefit from using more open-ended questioning in questionnaires designed to assess MHL.

## Conclusions

Our study provides updated findings on public knowledge about BED. Understanding what people do not know about BED is essential for the development of effective MHL initiatives. BED MHL initiatives are necessary to improve problem recognition, help-seeking, and access to effective treatments [2, 4] as well as to reduce the stigmatization of BED [2, 3].

## Supplementary Information

**Additional file 1.** MHL vignette and questionnaire.

## Data Availability

The data and materials supporting the conclusions of this article are available from the authors upon reasonable request.

## Abbreviation

BED MHL: Binge-eating disorder mental health literacy
