# Supplementary material for: A vignette study of mental health literacy for binge-eating disorder in a self-selected community sample
Source: J Eat Disord. 2023 May 4;11:69. doi: 10.1186/s40337-023-00795-y (PMC10161539; doi:10.1186/s40337-023-00795-y)
Supplement: Supplementary file 1 — Additional file 1. MHL vignette and questionnaire. [file 40337_2023_795_MOESM1_ESM.docx]

**Supplementary Materials**

**Article:**

A Vignette Study of Mental Health Literacy for Binge-Eating Disorder in a Self-Selected Community Sample

**Journal:**

Journal of Eating Disorders

**Authors:**

Kayla B. Hollett, Jenna M. Pennell, and Jacqueline C. Carter

Department of Psychology, Memorial University of Newfoundland, 230 Elizabeth Avenue, St. John’s, Newfoundland and Labrador, Canada, A1C 5S7

**Correspondence:**

Jacqueline C. Carter, jacquelinec@mun.ca

**BED Vignette**

Jane is a 30-year-old female with a full-time job. Jane eats three balanced meals every day and usually a snack or two. Every day when Jane gets home from work, she and her husband prepare supper that they eat at around 6:00PM. Later in the evening Jane usually goes to the kitchen to have a snack before she goes to bed. However, sometimes she finds that she is unable to stop eating after having the snack and continues to eat a large amount of food even though she is not hungry. For example, she may eat a peanut butter and jelly sandwich, a pack of cookies, two bowls of ice cream, and some chips all in one sitting. Jane is ashamed of this behaviour and eats the food secretly when her husband is not in the same room. During these times Jane feels out of control of her eating and often continues to eat until she feels uncomfortably full. After these episodes of eating Jane experiences feelings of disgust and guilt. She feels very distressed by these episodes, but she has never tried to compensate for what she has eaten (e.g., by fasting, vomiting, or using laxatives).

**Mental Health Literacy Questionnaire**

Please answer the following questions about the character you just read about, Jane. Your responses are completely anonymous and confidential. There are no right or wrong answers.

Please indicate which weight category you think Jane likely belongs to:

Underweight (BMI less than 18.5)

Recommended weight (BMI between 18.5 to 24.9)

Overweight (BMI between 25.0 to 29.9)

Moderately Obese (BMI between 30.0 and 34.9)

Severely obese (BMI between 35.0 and 39.9)

Do you think Jane has a problem?

Yes

No

Not sure

How would you describe Jane’s problem?

Physical health problem

Mental health problem

Both a physical and mental health problem

Other (please specify): __________

I do not think Jane has a problem

What would you say is Jane’s *main* problem? Carefully read all of the following options:

Depression

Anxiety

Bulimia nervosa

Anorexia nervosa

An eating disorder, but not anorexia or bulimia

Binge eating disorder

Obesity/overweight

Other (please specify): __________

Not sure

I do not think Jane has a problem

What do you think is the *primary cause* of Jane’s problem?

Biological factors (e.g., genetics)

Social factors (e.g., upbringing, life stressors)

Psychological factors (e.g., self-esteem)

Other (please specify): __________

Not sure

Not applicable (I do not think Jane has a problem)

Do you think that Jane should seek professional help for her problem?

Yes

No

Not applicable (I do not think Jane has a problem)

If Jane did receive help, which of the following person-based interventions do you believe would be most helpful? Please select up to 3 choices that you think would be most helpful for Jane.

Commercial weight-loss program (e.g. weight watchers)

Self-help support group (e.g., overeaters anonymous)

Family doctor (i.e., general practitioner)

Psychologist (i.e., a mental health care professional who specializes in the treatment of mental disorders through emotional and behavioural interventions)

Psychiatrist (i.e., a doctor who specializes in the treatment of mental disorders by prescribing medication)

Social worker or counsellor

Dietician (i.e., an expert in human nutrition)

Naturopath or another alternative therapist

Family member or close friend

Other (please specify): __________

Not sure

None of the above

Not applicable (I do not think Jane has a problem)

If Jane did receive help, which of the following therapy-based interventions do you believe would be most helpful? Please select up to 3 choices that you think would be most helpful for Jane.

Admission to a psychiatric ward

Cognitive behaviour therapy

Other psychotherapy (e.g., compassion focused therapy, acceptance and commitment therapy)

Using a self-help treatment manual or self-help book

Alternative therapy (e.g. naturopathy, homeopathy, etc.)

Family counselling/therapy

Behavioural weight loss and/or exercise program

Trying to deal with the problem on her own

Finding some new hobbies

Other (please specify): __________

Not sure

None of the above

Not applicable (I do not think Jane has a problem)

If Jane did receive help, which of the following medication-based interventions do you believe would be most helpful? Please select up to 3 choices that you think would be most helpful for Jane.

Vitamins and minerals

Antidepressant medication (e.g. Prozac or Zoloft)

Antianxiety medication (e.g. Valium or Serepax)

Herbal medicines/tonics

Other (please specify): __________

Not sure

None of the above

Not applicable (I do not think Jane has a problem)

What do you think Jane’s likely prognosis would be if she received treatment?

She would have a full recovery with no further problems

She would have a full recovery, but problems might reoccur

She would have a partial recovery

She would have no improvement

She would get worse

Not sure

Not applicable (I do not think Jane has a problem)

What do you think Jane’s likely prognosis would be if she did not receive treatment?

She would have a full recovery with no further problems

She would have a full recovery, but problems might reoccur

She would have a partial recovery

She would have no improvement

She would get worse

Not sure

Not applicable (I do not think Jane has a problem)

Do you believe you might currently have a problem similar to Jane’s problem?

Yes

No

In the past, have you experienced something similar to Jane’s problem?

Yes

No

**PAGE BREAK**

The character in the vignette, Jane, has binge eating disorder. Have you ever heard of binge eating disorder?

Yes

No

Do you think binge eating disorder is an official mental disorder?

Yes

No

Not sure

How common do you think binge eating disorder is in the general adult population?

0% of the population

Less than 1% of the population

1-5% of the population

5-10% of the population

10-15% of the population

15-20% of the population

More than 20% of the population
